# Knockout of elF4E using CRISPR/Cas9 for large-scale production of resistant cucumber cultivar against WMV, ZYMV, and PRSV

**DOI:** 10.3389/fpls.2023.1143813

**Published:** 2023-03-17

**Authors:** Hakan Fidan, Ozer Calis, Esin Ari, Aydin Atasayar, Pelin Sarikaya, Mumin Ibrahim Tek, Ahmet Izmirli, Yasemin Oz, Gulsah Firat

**Affiliations:** ^1^ Plant Protection Department Faculty of Agriculture Akdeniz University, Antalya, Türkiye; ^2^ Agricultural Biotechnology Department, Faculty of Agriculture, Akdeniz University, Antalya, Türkiye; ^3^ Research and Development Department AD ROSSEN Seeds, Antalya, Türkiye

**Keywords:** cucumber, CRISPR/Cas9, virus resistance, WMV, ZYMV, PRSV, gene-editing, tissue culture

## Abstract

CRISPR/Cas9 is one of the most robust technologies for plant breeding enabling precise and efficient modifications in a genome. This technology is being used for the manipulation of target genes in a host to develop resistance against the plant pathogens. *Cucumis sativus elF4E* is one of the target genes playing a key role in viral infection during interaction with potyvirus viral proteins genome linked (VPg). Nevertheless, the allelic and positional effect of *elF4E* mutations in *C. sativus* is to be clarified in *elF4E*-VPg interaction. In addition, there are entanglements in the massive production of pathogen-resistant cultivars suitable for commercial production using CRISPR/Cas9 technology. Therefore, we targeted different positions of the *elF4E* in G27 and G247 inbred lines, using specific gRNA1 and gRNA2 for the first and third exons, respectively, and 1,221 transgene-free plants were selected in segregated T1 generation, where 192 G27 and 79 G247 plants had the least mutation at Cas9 cleavage site of gRNA1 or gRNA2. Crossing was performed to see allelic effects of *elfF4E* mutations in F1 populations, which were homozygous and heterozygous single (elF4E_1^DEL^ or elF4E_3^DEL^) and double (elF4E_1-3^DEL^) mutants. Disease symptoms of watermelon mosaic virus (WMV), papaya ringspot virus (PRSV), and zucchini yellow mosaic virus (ZYMV) were evaluated in both non-edited and edited F1 plants, and we did not observe any symptom in homozygous elF4E_1-3^DEL^ and elF4E_1^DEL^ mutants. However, homozygous elF4E_3^DEL^ was positive in reverse transcription polymerase chain reaction (RT-PCR), even if there were no significant symptoms on the inoculated leaves. ELISA and qRT-PCR indicated lower viral accumulation in homozygous elF4E_3^DEL^ than heterozygous and non-edited plants. Regeneration and transformation protocols were also optimized comprehensively for both the genotypes. The average number of shoots/100 explants was determined for both G27 and G247 as 13.6 and 18.0, respectively. We could not detect any distinguishing difference between the non-edited and edited F1 plants for yield and morphology. Our results demonstrate an effective route for mass production of viral resistant cultivars of cucumber to WMV, ZYMV, and PRSV. In this way, the pathogen-resistant cultivars could be generated to reduce the losses caused by these pathogens in cucumber production.

## Introduction

Plant viruses are responsible for economic losses to agriculture production worldwide, with over 1,500 viruses belonging to 26 families (Cao et al., 2020). Members of the Potyviridae family, which includes zucchini yellow mosaic virus (ZYMV), papaya ringspot virus (PRSV), and watermelon mosaic virus (WMV), are detrimental pathogens particularly to cucurbit crops including cucumber. Potyviruses are single-positive stranded RNA viruses with relatively larger genome size, typically around 10 kb, than other plant pathogenic viruses (Revers and Garcia, 2015). Although various precautions such as sanitation are being implemented to control viral diseases, using virus-resistant cultivars is the most effective method to control them. However, traditional breeding approaches are inadequate and time consuming for developing resistant cultivars. Most of the dominant resistance (*R*) genes confer resistance against fungal and bacterial plant pathogens, rather than viruses (Truniger and Aranda, 2009; Wang and Krishnaswamy, 2012). On the other hand, several factors in the plant could facilitate the infection and increase susceptibility within host–virus interactions (Diaz-Pendon et al., 2004).

*Eukaryotic translation initiation factors* (*eIF*s) such as *elF4E* and *elF4G* have been extensively studied for their role in host–virus interactions in various plant species over the past two decades. It has been established that *elF4E* plays a key role in determining a host’s susceptibility or resistance to pathogenic viruses, despite its primary function as a regulator of cellular translation (Wang and Krishnaswamy, 2012). *elF4E* is known as the “cap-binding protein” and interacts with mRNA’s 5’-terminal cap and nuclear protein (Sonenberg and Gingras, 1998; Sonenberg and Dever, 2003). However viral proteins (VPg) encoded by viruses interact with *elF4E* by binding covalently to the host’s mRNA 5’-terminal cap (Murphy et al., 1996). Multiple research groups have demonstrated that VPg-*elF4E* interaction is essential for potyvirus infection, and loss of *elF4E* function confers recessive resistance against potyviruses in the host (Wittmann et al., 1997; Léonard et al., 2000; Ruffel et al., 2002). Naturally occurring mutants for *elF4E* variants have also been identified in plants, such as the *pvr2* allele in pepper, as well as controlled mutations that suppress *elF4E* function in plants (Ruffel et al., 2006).

Furthermore, identified *elF*s are not limited to *pvr2* allele in pepper; many *elF*s and their interactions were characterized in various studies. Most of the characterized recessive genes associated with the host–virus interactions are responsible for encoding *eIF4E*, *eIF4G*, and their isoforms. For instance, *pot-1* in tomato (*Solanum lycopersicum*), *rym4*, *rym5*, and *rym6* in barley (*Hordeum vulgare*), and *mo1* in lettuce (*Lactuca sativa*) were characterized as recessive genes encoding *elF4E* variants in plant–virus interactions (Nicaise et al., 2003; Kanyuka et al., 2005; Ruffel et al., 2006). The most critical characteristic of *elF4E* is responsible for susceptibility or resistance against viral pathogens, even if it contributes to cellular translation with its cellular translation function. Therefore, *elF4E*-mediated resistance is a valuable alternative to control plant viruses in agricultural production (Diaz-Pendon et al., 2004).

Developing new resistant cultivars through traditional breeding and introgression of *resistance (R*) genes from wild ancestors of commercial cultivars can be challenging, and pathogens can also overcome the *R*-gene–mediated resistance. Alternative approaches, such as using the loss of *susceptibility* (*S*) function mutants have been proposed to reduce host susceptibility. Some host proteins, known as S proteins, can increase infection rate and facilitate pathogen growth. Loss of *S* function can provide durable, broad-spectrum resistance in plants, because the viability of the obligate pathogens such as viruses depends on the host factors (de Almeida Engler et al., 2005; van Schie and Takken, 2014).

It has been demonstrated that homozygous *elF4E* mutations can confer resistance against potyviruses in various plant species. For example, deletion mutations in *Arabidopsis thaliana elF4E* and *elF(iso)4E* provide complete resistance to turnip mosaic virus (TuMV) without affecting plant vigor (Pyott et al., 2016). Induced deletion mutations in tobacco *elF4E* genes (*elF4E1-S*, *elF4E1-T*, *elF4E2-S*, and *elF4E2-T*) also conferred higher level of resistance to potato virus Y (PVY), another member of the potyvirus family (Le et al., 2022). Silencing of *elF4E* has shown broad-spectrum resistance against RNA viruses in tomato (Mazier et al., 2011), besides determining resistance to potyvirus in naturally occurring mutant plants for *elF4E* and *eIF(iso)4E* (Gómez et al., 2009). Additionally, cucumber vein yellowing virus (CVYV), ZYMV, and PRSV-resistant cucumber plants with homozygous substitutions and deletions in *elF4E* have been generated using CRISPR/Cas9 (Chandrasekaran et al., 2016).

The allelic and positional effects of *elF4E* mutations on potyvirus resistance remain unclear in *C. sativus*, despite previous reports of loss of *elF4E* function in various plants including cucumber. Additionally, the mass production of CRISPR/Cas9-edited plants resistant to plant pathogens has not been extensively studied. Therefore, we have conducted this study to investigate the positional and allelic effects of *elF4E* mutations in *C. sativus* and to demonstrate an effective method for generating pathogen-resistant mutant cultivars with CRISPR/Cas9, suitable for commercial use in agricultural production. We selected two inbred lines, G27 and G247, which were regenerated after transformation and determined their regeneration and transformation efficiencies based on the comprehensive optimization trials. Homozygous and heterozygous single and double non-transgenic mutants in T2 were used to determine the allelic and positional effects of *elF4E* mutations. We compared the F1 plants for agronomic value, morphology, and virus resistance. This allowed us to examine the loss of *elF4E* function in cucumber not only for potyvirus resistance but also for its effects on plant morphology and agronomic traits such as yield, fruit, and plant size.

## Results

### Transformation and regeneration protocol for G27 and G247 inbred lines

**Figure 1:**
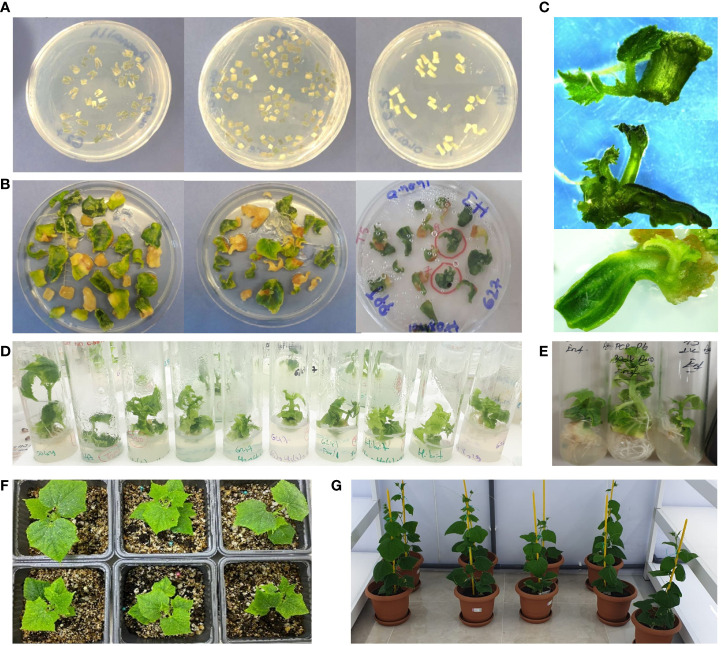
Plant regeneration from transformed cucumber cotyledon leaves. Cotyledon leaves are dissected into a base and two distal pieces **(A)**. The cotyledons, their proximal ends, and hypocotyl pieces are incubated in co-culture medium with *Agrobacterium tumefaciens* EHA105 cells with pFGC-pcoCas9 plasmid T-DNA vector **(B)**. Shoot formations from cotyledon leaves **(C, D)**. *In-vitro* plants ready for acclimatization **(E)**. Acclimatized cucumber regenerants **(F)**. Growth of the cucumber plants 1 month after potting in the greenhouse **(G)**.

Comprehensive protocol optimization of regeneration and transformation was performed for both G27 and G247 genotypes, with 30 transformation experiments. The most optimal conditions for the transformation of G27 and G247 were determined using EHA105 strain of *Agrobacterium tumefaciens*, 1-day-old seedlings (plant age), cotyledons with proximal ends as the explant type, and 300 mg l^-1^ timentin antibiotic in medium to suppress bacterial growth. Additionally, 1.5 mg l^-1^ BAP (6- benzylaminopurine) and 1.0 mg l^-1^ ABA (abscisic acid) were found the most effective in inducing shoot growth for both genotypes without preculture. Under these optimized conditions, the average number of shoots per 100 explants was 13.6 for G27 and 18 for G247 genotypes (**Supplementary Tables S1**, **S2**). The transformation efficiency of G247 was higher than G27. Following the acclimatization stage, a total of 174 *ex-vitro* plants were transferred to a greenhouse (**Figure 1**). Meanwhile, PCR using Ag-CT0 primers was carried out for screening of 64 T0 plants to confirm T-DNA insertion into G27 and G247 inbred lines. Among these, 34 G27 and 22 G247 plants were found to be T-DNA positive and then we harvested T1 seeds from these plants.

### CRISPR-induced mutations of G27 and G247 in T1 generation

The screening was performed in T1 population; 2,315 plants of G27 and 1,639 plants of G247 were T-DNA positive, while 751 of G27 and 470 of G247 were transgene free. The number of transgene-free plants was evaluated with the chi-square test, and results were fit with Mendelian segregation (**Supplementary Table S3**). A total of 357 T1 plants composed of 251 of G27 and 106 of G247 were also screened to detect homozygous or heterozygous mutant plants with PCR using MC1F/R and MC2F/R primers and digested with *MvaI* and *PsuI* restriction enzymes. The results indicated that 192 of G27 and 79 of G247 had the least mutation at Cas9 cleavage site of gRNA1 or gRNA2 position on *elF4E*. The total number of homozygous mutations to gRNA1 was 42 for G27 and 16 for G247, which were higher than the gRNA2 position. Most of the mutations were heterozygous at Cas9 cleavage site of gRNA2 in G27 and G247, and their number was 39 for G27 and 16 for G247. Furthermore, gRNA1 and gRNA2 positions of selected 40 transgene-free plants in G27 and G247 were amplified with MC1F/R and MC2F/R primers in PCR followed by the Sanger sequencing to determine insertion, deletion, and substitution caused by non-homologous end joining (NHEJ). The mutation types were determined at the gRNA1 position in 21 plants and the gRNA2 position in five plants, respectively. The largest deletions were detected in gRNA1 of G27-M98 with 5 bp deletion, and 1 bp deletion was the smallest in G27-M70. The most common deletions were 2 bp and were detected on G27-M36, G27-254, G27-M485, G247-M4464, and G247-M4591 for gRNA1. The deletions have appeared at gRNA2, and were maximum of 4 bp for G27-M36 and G247-M4464, and minimum of 2 bp for G27-MB7 and G247-M4591 (**Supplementary Figure S2**). Although there was no insertion mutation at the gRNA1 or gRNA2 cleavage site of Cas9 in sequenced T1 plants, 1 bp (C/T) transition mutation was detected on the gRNA1 position of G27-M98 (**Supplementary Table S4**).

### F1 populations were generated to observe positional and allelic effects of mutations

T2 seeds were harvested from T1 G27 and G247 mutant plants, and selected T2 plants were used for crossing combinations to generate F1 populations (**Table 1**). The G27-M36 and G247-M4464 had 2 and 4 bp deletion at the Cas9 cleavage site of gRNA1 and gRNA2, respectively, and they were used to generate homozygous elF4E_1-3^DEL^ F1 populations, while as G27-M36 was crossed with non-edited G247 to obtain heterozygous elF4E_1-3^DEL^ F1 mutants. The G27-254 and G247-M4591 had 2 bp deletions at the gRNA1 site, so they were crossed to obtain homozygous elF4E_1^DEL^ F1 plants, and G27-254 was used in crossing combinations with non-edited G247 for heterozygous elF4E_1^DEL^ genotype. Similarly, G27-MB7 and G247-M398 that had two deletions at the gRNA2 position were used to generate homozygous elF4E_3^DEL^ F1 populations, and G27-MB7 was crossed with non-edited G247 for heterozygous elF4E_3^DEL^. Their F1 genotypes were confirmed with *MvaI* and *PsuI* restriction enzymes after amplification of gRNA targets using PCR with MC1F/R and MC2F/R primers.

**Table 1:** Crossing plots to generate different F1 genotypes.

	G27-M36 × G247-M4464		G27-M36 × G247-NE	
**gRNA1**	2 del	2 del	2 del	WT
**gRNA2**	4 del	4 del	4 del	WT
**F1**	*Homozygous elF4E_1-3^DEL^*		*Heterozygous elF4E_1-3^DEL^*	
	G27-254 × G247-M4591		G27-254 × G247-NE	
**gRNA1**	2 del	2 del	2 del	WT
**gRNA2**	WT	WT	WT	WT
**F1**	*Homozygous elF4E_1^DEL^*		*Heterozygous elF4E_1^DEL^*	
	G27-MB7 × G247-M398		G27-MB7 × G247-NE	
**gRNA1**	WT	WT	WT	WT
**gRNA2**	2 del	2 del	2 del	WT
**F1**	*Homozygous elF4E_3^DEL^*		*Heterozygous elF4E_3^DEL^*	

### Deletions causing amino acid alteration and stop codon formation in edited F1

**Figure 2:**
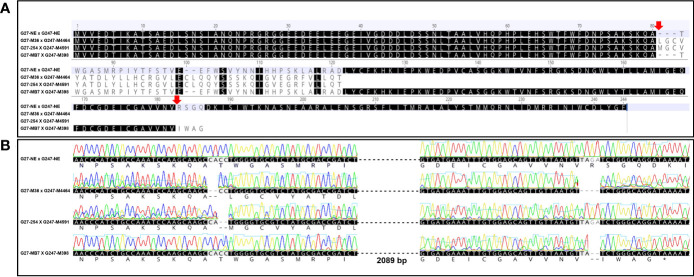
Alignment of the amino acid sequences of non-edited and edited F1s. Red arrows indicate the Cas9-cleavage sites at the gRNA1 and gRNA2 **(A)**. Alignment of the nucleotide and amino acids sequences of edited and non-edited lines with chromatogram data. G27-M36 × G247-M4464 and G27-254 × G247-M4591 amino acids sequences at gRNA2 positions were not given because of the early stop codon formation at the 122^nd^ position of elF4E amino acid sequence **(B)**.

The same genotypes of induced mutation type by Cas9 cleavage of *elF4E* have been selected for crossing plots to prevent chimerism in F1 populations (**Table 1**). Edited F1’s amino acid and nucleotide sequences of *elF4E* were aligned with non-edited F1 plants (**Figure 2**). However, there was no difference between the amino acid sequence of G27-M36 × G247-M4464 and G27-254 × G247-M4591, even if they are different type of mutants as well as double and single mutants according to nucleotide sequence, respectively. The alteration was started at the 80^th^ amino acid of *elF4E*, which was the Cas9 cleavage site of gRNA1 for G27-M36 × G247-M4464 and G27-254 × G247-M4591. The stop-codon formation was detected at the 116^th^ amino acid for G27-M36 × G247-M4464 and G27-254 × G247-M4591. Another single mutant F1 for the gRNA2 position of *elF4E* was generated with the crossing of G27-MB7 and G247-M398, which had 2 bp deletions. The deletion caused stop-codon formation at the 181^st^ amino acid position, after the altered amino acids “IWAG” rather than “RSGQ” (**Figure 2**).

### Homozygous elF4E_1-3^DEL^, elF4E_1^DEL^, and elF4E_3^DEL^ did not show symptoms associated with inoculated viruses

**Figure 3:**
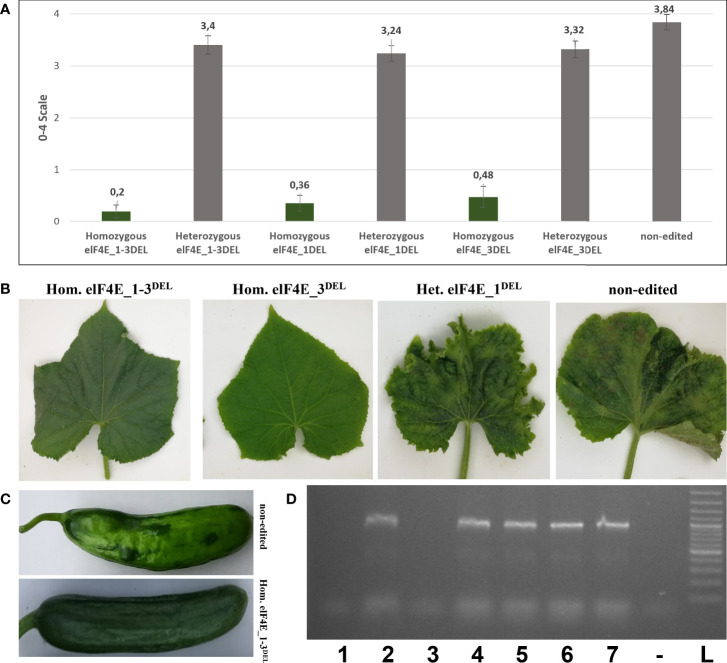
Evaluation of zucchini yellow mosaic virus (ZYMV) inoculation in edited and non-edited F1 plants. Average scores of ZYMV-inoculated plants for non-edited F1s, homozygous mutants, and heterozygous mutants. The same column color indicates that there is no statistical difference between groups **(A)**. ZYMV symptoms on homozygous elF4E_1-3^DEL^ were absent, while restricted lesions were detected on 1-2 leaves of homozygous elF4E_3^DEL^, heterozygous elf4E_1^DEL^, and non-edited F1s displayed typical symptoms **(B)**. ZYMV-associated symptoms on homozygous elF4E_1-3^DEL^ and non-edited fruits **(C)**. RT-PCR results showed that only homozygous elF4E_1-3^DEL^ (lane 1) and homozygous elF4E_1^DEL^ (lane 3) mutants were negative. However, homozygouelF4E_3^DEL^ (lane 5) was positive in RT-PCR, similarly non-edited (lane 7), and heterozygous mutants **(D)**.

**Figure 4:**
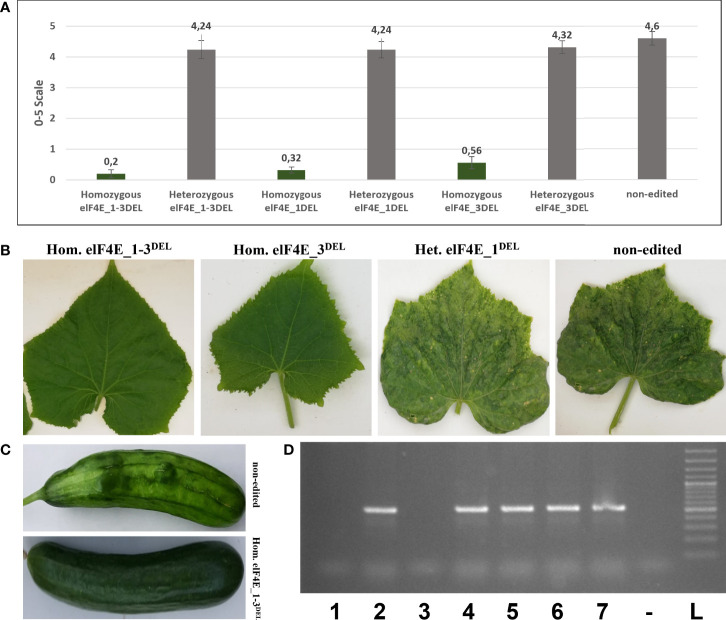
Evaluation of papaya ringspot virus (PRSV) resistance in edited and non-edited F1 plants. Disease scores on a 0–4 scale, the average scores were presented with standard deviation bars, and the same colors are indicating that there is no significant difference between evaluated groups **(A)**. PRSV symptoms on leaves and fruit were seen on non-edited and heterozygous mutant leaves and fruits, while there were no symptoms on homozygous elF4E_1-3^DEL^, homozygous elF4E_1^DEL^, and homozygous elF4E_1-3^DEL^ **(B, C)**. Homozygous elF4E_1-3^DEL^ (lane 1) and homozygous elF4E_1^DEL^ (lane 3) were negative in RT-PCR, while others (lanes 2, 4, 6, and 7) including homozygous elF4E_3^DEL^ (lane 5) were positive **(D)**.

**Figure 5:**
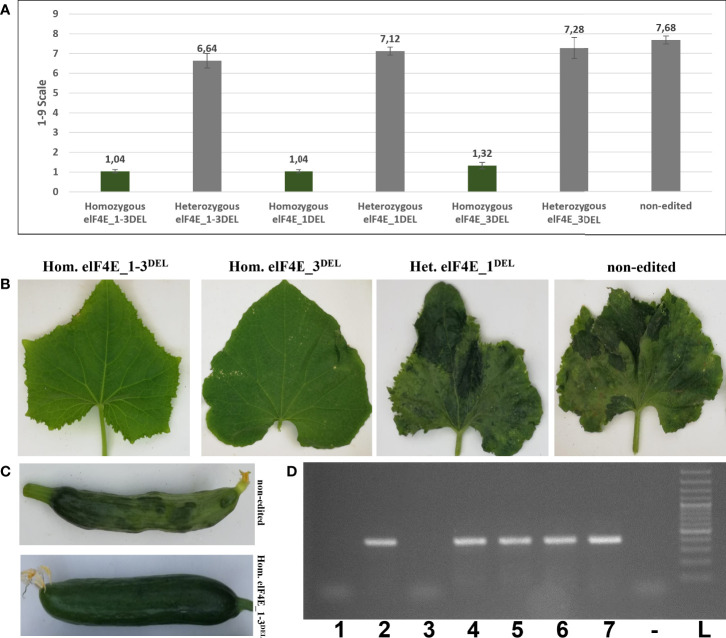
Assessment of watermelon mosaic virus (WMV) inoculation in edited and non-edited F1 plants. Disease scores were rated according to the 0–5 scale, and the groups that have the same color column are indicating statistically indistinguishable in graphic **(A)**. WMV symptoms on leaves were observed 12 days after inoculation, and typical symptoms were detected on non-edited F1 fruit **(B, C)**. Homozygous elF4E_3^DEL^ mutant (lane 5) was positive in RT-PCR, like heterozygous and non-edited F1s, even though there were no symptoms on homozygous elF4E_3^DEL^ leaves or fruit. Homozygous elF4E_1-3^DEL^ (lane 1) and homozygous elF4E_1^DEL^ (lane 3) were negative according to RT-PCR **(D)**.

The leaves of generated F1 plants including control non-edited F1 plants and resistant plants were inoculated with WMV, ZYMV, and PRSV; each genotype was evaluated according to 0–5, 1–9, and 0–4 scales, respectively (Wai and Grumet, 1995; Guner et al., 2002; Palomares-Rius et al., 2011). The WMV and ZYMV symptoms appeared on non-edited F1 plants at 7 dpi, while PRSV symptoms were detected on leaves of non-edited F1 at 20 dpi. Evaluation of the virus symptoms of non-edited plants using different scales revealed: 4.60 for ZYMV (**Figure 3**), 7.68 for PRSV (**Figure 4**), and 3.84 for WMV (**Figure 5**). The results were similar with the heterozygous elF4E_1-3^DEL^, elF4E_1^DEL^, and elF4E_3^DEL^ F1 plants. Their scores were WMV = 4.24, ZYMV = 6.64, and PRSV = 3.4 for heterozygous elF4E_1-3^DEL^; WMV = 4.24, ZYMV = 7.12, and PRSV = 3.24 for heterozygous elF4E_1^DEL^; WMV = 4.32, ZYMV = 7.28, and PRSV = 3.32 for heterozygous elF4E_3^DEL^. We did not find any resistance reaction in heterozygous mutations in these genotypes. However, the lowest scores were measured in homozygous mutants for elF4E_1-3^DEL^, elF4E_1^DEL^, and elF4E_3^DEL^. There were no symptoms in homozygous mutants elF4E_1-3^DEL^ and elF4E_1^DEL^ F1 plants for each inoculated virus, while as restricted small lesions associated with ZYMV were detected on 1-2 leaves of homozygous elF4E_3^DEL^ F1s.

### Homozygous elF4E_1-3^DEL^ and elF4E_1^DEL^ plants that were negative in reverse transcription polymerase chain reaction and enzyme-linked immunosorbent assay

**Figure 6:**
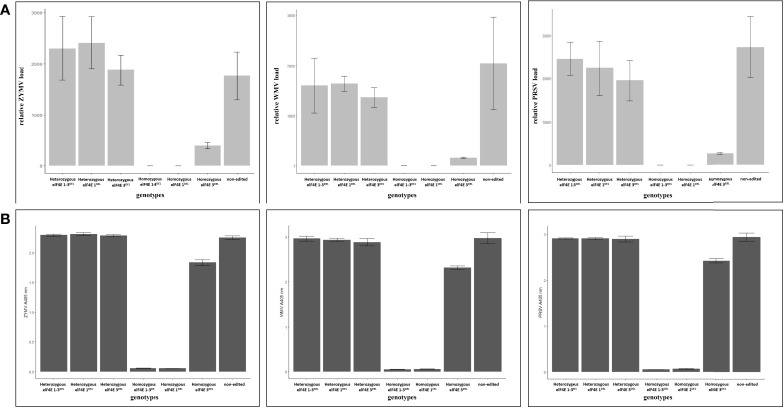
Determination of viral accumulation in edited and non-edited F1 plants according to 2^−ΔΔCT^ and absorbance at 405 nm, derived from quantitative reverse transcription polymerase chain reaction (qRT-PCR) and DAS-ELISA, respectively. There were no significant differences between the non-edited and heterozygous plants for viral accumulation for each inoculated virus. However, lower viral accumulation was detected in homozygous elF4E_3^DEL^ relative to non-edited and heterozygous mutants. Also, the accumulation of each virus was higher in homozygous elF4E_3^DEL^ than homozygous elF4E1_3^DEL^ and homozygous elF4E1^DEL^ **(A)**. Absorbance values of samples were parallel to relative viral accumulation results derived from the qRT-PCR for edited and non-edited F1s **(B)**.

The leaves were harvested from inoculated F1 plants including edited as well as control non-edited plants, and the samples were purified and prepared for reverse transcription polymerase chain reaction (RT-PCR), quantitative RT-PCR (qRT-PCR), and enzyme-linked immunosorbent assay (ELISA). The results have shown that homozygous elF4E_1-3^DEL^ and elF4E_1^DEL^ plants were negative in RT-PCR, while other heterozygous and non-edited plants were positive for ZYMV (**Figure 3**), PRSV (**Figure 4**), and WMV (**Figure 5**). However, homozygous elF4E_3^DEL^ was positive in RT-PCR, while they did not show any symptoms after the WMV, ZYMV, and PRSV inoculation. Viral loads were also evaluated with qRT-PCR and ELISA for inoculated non-edited and edited F1s. ELISA results were determined as positive for homozygous elF4E_3^DEL^, and only homozygous elF4E_1-3^DEL^ and elF4E_1^DEL^ plants were negative in ELISA. The absorbance values at 405 nm of WMV, ZYMV, and PRSV inoculated plants indicated that the viral load of homozygous elF4E_3^DEL^ was less than heterozygous and non-edited F1s. We did not detect any viral loads in homozygous elF4E_1-3^DEL^ and elF4E_1^DEL^ plants according to qRT-PCR. However, relative ZYMV, WMV, and PRSV loads were higher in homozygous elF4E_3^DEL^ than homozygous elF4E_1-3^DEL^ and elF4E_1^DEL^ plants, even if viral load of homozygous elF4E_3^DEL^ was lower than heterozygous mutants and non-edited plants (**Figure 6**).

### Yield and morphological difference in edited and non-edited F1 plants

**Figure 7:**
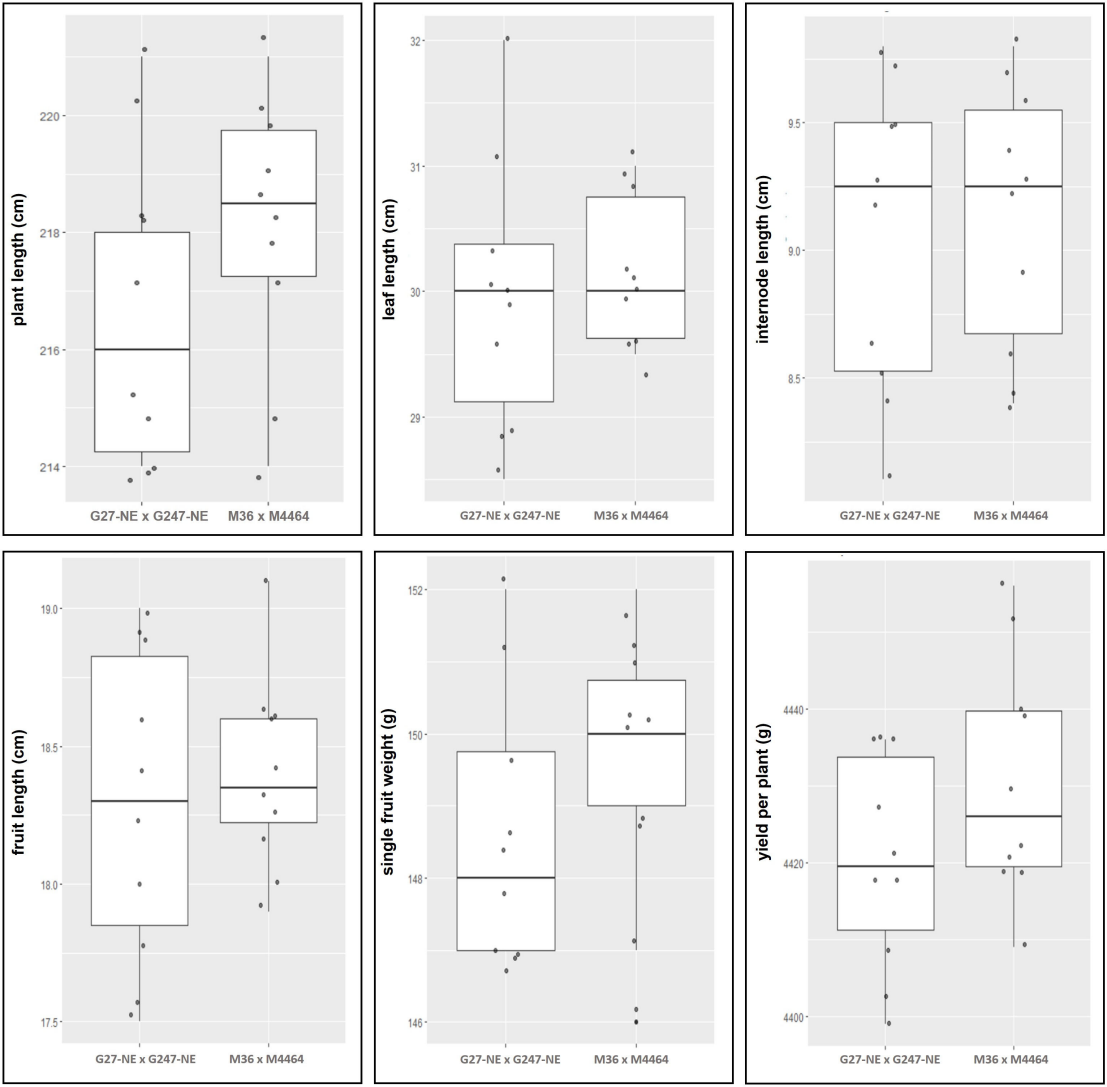
Comparison of morphological and yield characteristics between non-edited (G27-NE and G247-NE) and homozygous elF4E_1-3^DEL^ (G27-M36 × G247-M4464) plants were given in graphs. No statistically significant differences were observed between the two populations according to Welch Two Sample *t*-test (*p* = 0.05). Boxplots are created using data, given in **Supplementary Table S5**.

The edited F1s and non-edited F1 populations were compared for various morphological criteria including internode, plant, leaf, fruit lengths, yield, single fruit weight, and yield per plant. The homozygous elF4E_1-3^DEL^ F1 (G27-M36 × G247-M4464) and non-edited F1 (G27-NE × G247-NE) were used for comparison at the harvesting period and measured criteria were given in **Supplementary Table S5**. There was no statistically significant difference (*p* = 0.05) in the compared groups in terms of internode, leaf, and fruit lengths according to Welch Two Sample *t*-test (**Supplementary Table S6**). We did not observe any yield penalties in homozygous elF4E_1-3^DEL^ F1 (**Figure 7**).

## Discussion

The climate crisis is having a significant impact on plant health and the spread of pathogens. Higher temperatures and changing weather patterns make plants more vulnerable to infections that can cause significant damage to THE crop and natural vegetation. Viral diseases can greatly reduce agricultural production, leading to economic losses and food insecurity. Using traditional breeding methods to develop virus-resistant plants is not sufficient in the face of rapidly evolving viruses and the challenges posed by the climate crisis. Classical methods can be time-consuming, imprecise, and limited in generating new disease-resistant cultivars. Thus, CRISPR/Cas9 and other gene-editing methods offer a solution to generate new resistant plants to viral, bacterial, and fungal pathogens (Karavolias et al., 2021).

In this study, we focused on the manipulation of *elF4E*-Vpg interaction, a key requirement for potyvirus infection in host plants, to generate the loss-of-*elF4E* function mutant for *C. sativus*. It has been reported that manipulation of the *elF4E*-Vpg interaction confers broad-spectrum potyvirus resistance in various plants, including cucumber (Chandrasekaran et al., 2016; Macovei et al., 2018; Gomez et al., 2019). Boosted potyvirus virus resistance was also reported in various plants using CRISPR/Cas9 and homozygous plants showed resistance after inoculation of tungro spherical virus in rice or cassava brown streak virus in cassava (Macovei et al., 2018; Gomez et al., 2019). Our aim was not only to investigate the resistance mediated by loss of *elF4E* function in cucumber but also to determine the positional and allelic effects of *elF4E* mutations in cucumber. To this end, we constructed two guide RNAs targeting exon 1 and exon 3 of *C. sativus elF4E* to generate different types of mutants. Homozygous elF4E_1-3^DEL^ double mutants, homozygous elF4E_1^DEL^ single mutant, and elF4E_3^DEL^ single mutants were identified in the T1 generation from transgene-free plants, and T2 seeds harvested after self-crossing. We used the G27-M36, G247-M4464, G27-254, G247-M398, G247-M4591, G27-MB7, G27-NE, and G247-NE inbred lines in crossing experiments. Homozygous and heterozygous F1s were produced for double and single *elF4E* mutations. We followed the same path with previously reported research by Chandrasekaran et al. (2016) and the gRNA2 was different by only one bp than their study. However, their gRNA1 (exon 1 target) position did not hit our genotypes’ *elF4E* and when we blast in Pyhtozome, results hit with the *Cucsa.300080*. Hence, we have selected both gRNAs from the sequence of *Cucsa.212630*.

WMV, ZYMV, and PRSV were inoculated onto each F1 plants, including both edited and non-edited F1 genotypes, through both mechanical and aphid transmission. The virus symptoms indicated that homozygous elF4E_1-3^DEL^, elF4E_1^DEL^, and elF4E_3^DEL^ mutants are resistant to WMV, ZYMV, and PRSV. Previously, *elF4E*-mediated resistance to ZYMV and PRSV is reported for mechanical inoculation and aphid-transmitted viruses in greenhouse conditions. However, *elF4E*-mediated WMV resistance was not reported in *C. sativus*. Homozygous elF4E_1-3^DEL^ and homozygous elF4E_1^DEL^ F1s showed resistance not only to mechanically inoculated viruses but also to aphid-transmitted ZYMV, WMV, and PRSV. While as the quite limited ZYMV and WMV symptoms were detected on homozygous elF4E_3^DEL^ F1 plants, these symptoms were less severe than in non-edited F1s and other heterozygous single and double mutants for *elF4E*. Additionally, homozygous elF4E_3^DEL^ mutant F1 plants are positive in RT-PCR, even though fewer viral symptoms were observed on their leaves. It could be clarified with the viral load of WMV, ZYMV, and PRSV. ELISA and qRT-PCR results showed that the relative viral loads in homozygous elF4E_3^DEL^ were higher than in homozygous mutants, even if lower than in non-edited and heterozygous mutants.

The *elF4E*-binding domain of VPgs in potyviruses has been reported, and the function of this domain is thought to be responsible for potyvirus replication. Identification of *elF4E* function in lettuce has indicated *elF4E* interact with VPg or host and pathogen factors and their mechanisms could involve intracellular and cell-to-cell trafficking or encapsidation (German-Retana et al., 2008). Also, *elF4E*’s function was associated with the potyvirus cell-to-cell movement in pea and pepper (Gao et al., 2004). However, the binding target of VPg is not well understood in *C. sativus*, but we speculate that the specific target of VPg may be the second or third exon of *elF4E*. This is because mutations in the first exon that result in an amino acid alteration confer complete resistance, as indicated by negative results in RT-PCR and ELISA tests in these plants. These findings are consistent with those observed in homozygous elF4E_1-3^DEL^ and elF4E_1^DEL^. However, heterozygous mutations in gRNA1 and gRNA2 locations did not result in resistance in F1 plants. These plants showed common symptoms upon inoculation with mechanically or aphid transmitted WMV, PRSV, and ZYMV, and the scores of these inoculated heterozygous mutants are not different from those of non-edited F1s. Also, WMV, ZYMV, and PRSV can multiply in the host even when mutations occur in the third exon of *elF4E*, as in homozygous elF4E_3^DEL^.

Furthermore, susceptibility was reduced in single elF4E_1^DEL^ homozygous mutant F1s against WMV, ZYMV, and PRSV. qRT-PCR and ELISA results indicated that WMV, ZYMV, and PRSV cannot multiply in double elF4E_1-3^DEL^ and single elF4E_1^DEL^ mutant F1 plants, because there was no viral accumulation in elF4E_1-3^DEL^ and single elF4E_1^DEL^. Although the viral loads and virus-associated symptoms were lower in homozygous elF4E_3^DEL^ than heterozygous mutants and non-edited plants, the viral accumulations were detected for each virus with ELISA and qRT-PCR in homozygous elF4E_3^DEL^. The results strongly suggest that VPgs of potyviruses are associated with the first or second exon of the *C*. *sativus elF4E*. However, further experiments are required to determine the function of the elF4E protein and its functional domains in the cucumber for the understanding of the potyviruses-plant interactions.

Elongation factors are also called cap-binding proteins, because their regulator functions in cellular translation with the interaction of mRNA’s 5’-terminal cap and nuclear proteins (Sonenberg and Gingras, 1998; Sonenberg and Dever, 2003). However, the loss of *elF4E* function did not affect plant morphology or other yield characteristics in homozygous elF4E_1-3^DEL^. Welch Two Sample *t*-test showed that there was no significant difference between edited and non-edited F1 plants for plant length, leaf length, internode length, fruit length, yield per plant, and single fruit weight. Similarly, deletion mutations on *elF4E* and *elF(iso)4E* did not change plant vigor in *Arabidopsis thaliana*, besides deletions confer the resistance against TuMV (Pyott et al., 2016). Additionally, naturally occurring mutants for *elF4E* or *elF(iso)4E* were characterized for potyvirus resistance in various plants (Gómez et al., 2009). Studies on the function of *elF4E* are mostly related to its potyvirus interactions. Potyviruses have covalently bonded VPg at 5′ of their RNAs, rather than a 7-methylguanosine cap like in some eukaryotic and viral mRNAs. Therefore, elF4E or *elF(iso)4E* interaction is essential for VPg to avoid RNA silencing and translation or stabilization of viral structures (Saha and Mäkinen, 2020). The role of *elF4E* has also been reported for lettuce mosaic virus infection cycle in lettuce (*Lactuca sativa*), and it has been suggested that *elF4E* function could be different from cellular mRNA translation (German-Retana et al., 2008). Hence, controlled mutations to suppress the function of *elF4E* function with novel gene-editing methods could be a reliable and robust way to generate new potyvirus-resistant cultivars without yield penalties.

Although CRISPR/Cas9 technology has been demonstrated as a reliable means of precisely modifying plant genomes, there are technical challenges that must be addressed to use it for the commercial production of pathogen-resistant cultivars. It is important to consider both scientific knowledge and commercial production requirements to effectively utilize CRISPR-modified plants in agriculture. Regeneration steps are essential for developing gene-edited crops after the *Agrobacterium*-mediated or particle bombardment transformation. The regeneration process is one of the most entanglements for gene-editing methods for non-model plants (Shan et al., 2020), because efficient and successful regeneration is crucial for both transgenic studies and the production of gene-edited crops by using CRISPR. Poor regeneration and transformation rates can impede genetic transformation efforts (Tan et al., 2022). The regeneration rate is largely influenced by the genotype and various factors such as the source of explants, seedling stage (Chang et al., 2018), exogenous hormones (Tang et al., 2010; Tang et al., 2011; Hiroshi et al., 2016), *Agrobacterium* strains (Zheng, 2009), the pre-culture period (Wang et al., 2014), and selection markers (Li et al., 2014). Therefore, optimization of protocols for the G27 and G247 inbred lines was performed before attempting the transformation and regeneration of T0 plants. The most effective regeneration was observed in T29, with 33.01 and 33.68 shoots induced for G27 and G247, respectively.

The comparison of the transformation efficiency for *Agrobacterium* strains showed that EHA105-mediated transformation is more effective than LBA4404-mediated transformation in cotyledon and hypocotyl explants for both genotypes. Hypocotyl explants did not induce shoot growth in either LBA4404- or EHA105-mediated transformation in G27. The efficiency of various *A. tumefaciens* strains and explant sources has been reported, and efficiencies ranging were 0.89% to 21% for EHA105 and 0.5% to 4.8% for LBA4404. In this study, EHA105 was found to have a higher transformant shooting rate than LBA4404. The effect of seedling age on regeneration was also evaluated, and it was found that using 1-day-old seedlings’ cotyledons resulted in a higher number of regenerated shoots in both G27 and G247, compared with using 5-day-old seedlings. This finding is consistent with previous reports that younger cucumber explants lead to higher regeneration efficiency (Chang et al., 2018). The optimal concentration of ABA and BAP for shoot induction was found to be 1.5 mg l^-1^ of both ABA and BA for both G27 and G247. Liu et al. (2018) also found that a regeneration frequency of 96.7% was achieved using 1.5 mg l^-1^ BAP and 1.0 mg l^-1^ ABA in their genotypes (variety 9330). Following the optimization of transformation and regeneration, T0 plants were obtained and verified using PCR. T1 seeds were harvested from the regenerated transgenic plants, and T2 populations were generated from transgene-free T1 plants for each genotype. To avoid chimerism and heterozygosity in the F1 generation, plants from T2 G27 and G247 that have the same mutation at the gRNA target site were selected. These plants were self-crossed and crossed with another parental line to produce and store seeds of loss-of-function mutants. The G27-M36 and G247-M4464 inbred lines were used in crossing combinations, resulting in edited resistance F1 plants against WMV, ZYMV, and PRSV.

In conclusion, we have used CRISPR/Cas9 to reduce susceptibility in G27 and G247 cucumber inbred lines by suppressing *elF4E* gene, which is associated with potyviruses. The positional and allelic effects of the *elF4E* mutations were investigated in different heterozygous or homozygous and single or double mutants. We found that stop-codon formation at the second exon of *elF4E* and amino acid substitutions conferred complete resistance to WMV, PRSV, and ZYMV without any viral load. G27M36 and G247M4464, which have 4 and 2 bp deletion mutations at *elF4E*, were crossed to produce F1 plants. These edited F1 plants were compared with non-edited F1 plants, and no significant differences were observed in terms of yield, quality, or morphology. In addition, we have also optimized transformation and regeneration protocols for *C. sativus* G27 and G247 inbred lines for large-scale production of the edited plants. This study contributes the potential of using CRISPR/Cas9 to generate resistant plants with knockout susceptibility genes for suitable commercial agricultural production. This approach could be used to generate a variety of resistant cultivars to manage viral, fungal, and bacterial plant pathogens in future.

## Materials and methods

### Plant materials

G27 (♀) and G247 (♂) cucumber inbred lines were used in this study as plant material. The non-edited F1 plants had the following characteristics: plant length of 210–225 cm, internode length of 8–10 cm, leaf length of 30–32 cm, fruit length of 17–19 cm, and single fruit weight of 140–150 g. These plants were not resistant to ZYMV, WMV, and PRSV, as they did not possess specific R genes such as *zym* and *wmv02245*.

### gRNAs and vector construction

*C sativus elF4E–*specific gRNA1 and gRNA2 were selected (**Supplementary Figure S1**) from the CRISPOR webtool (Concordet and Haeussler, 2018) and synthesized as oligonucleotides. These gRNAs were then individually consubstantiated in pTWIST Amp vector with the AtU6 promoter and scaffolds. XhoI : AtU6:gRNA1:scaffold-PacI and PacI : AtU6:gRNA2:scaffold:XbaI constructs were obtained using PCR to add the overhang of the restriction site. T4 DNA ligase was used to linearly ligate the two constructs, and then the reaction mixture was prepared with 1 µl of T4 DNA ligase, 2 µl of T4 DNA ligase buffer (10×), 12.5 µl of ddH2O, and 150-ng construct. The resulting ligated construct was assembled in the pFGC-pcoCas9 vector (Addgene: 52256) using restriction site cloning with *XhoI* and *XbaI*. The gRNA’s construct that was cloned into pFGC-pcoCas9 was confirmed with *XhoI/XbaI* restriction enzymes after T4 ligation. The cloned plasmid (**Supplementary Figure S1**) was then transferred to *E. coli* DH5α cells *via* heat shock, as previously described by Froger and Hall (2007). Transformant *E. coli* cells were selected on kanamycin (50 ng µl^-1^) containing Luria-Bertani Agar (LBA), and the plasmid was isolated from *E. coli* DH5α cells. The isolated plasmid was then transferred to *Agrobacterium tumefaciens* EHA105 electrocompetent cells using electroporation (Gene Pulser Xcell Electroporation System, BioRad, California, USA), and the cells were stored at -80°C after selection on kanamycin (50 ng µl^-1^), and rifampicin (15 ng µl^-1^) in LBA.

### Transformation and regeneration

Optimization trials were conducted to optimize regeneration and transformation in G27 and G247. Specifically, 50 different culture media were tested for regeneration, and a medium consisting of MS + 1.5 mg l^-1^ BA + 1.5 mg l^-1^ ABA was selected (data not shown). For transformation, various factors including genotype, bacterial strain, seedling age, explant type, pre-culture, and antibiotic were optimized to maximize transformation efficiency. A total of 30 transformation optimization trials were performed for each genotype, resulting in the identification of optimal conditions for the final eight transformations (**Supplementary Table S2**).

To surface disinfect, the seeds of G27 and G247 were first immersed in 70% ethanol for 1 min, then treated with a solution of 15% commercial bleach containing 0.05% Tween 20 for 15 min. Following this, the seeds were rinsed three times. These seeds were then germinated *in vitro* on a medium composed of MS salts (Murashige and Skoog, 1962), Nitsch and Nitsch vitamins (Nitsch and Nitsch, 1969), 3% sucrose, and 0.7% agar (pH 5.8) in the dark at 28°C for 1 day. Cotyledon explants were obtained from 1-day-old germinated seedlings by cutting their proximal regions using a liquid regeneration medium (MS medium supplemented with 3% sucrose and 200 µM acetosyringone). Meanwhile, to establish bacterial colony cultures of the strain EHA105 carrying the pFGC-pcoCas9 plasmid of *Agrobacterium tumafaciens*, bacterial cells were streaked onto solid Luria Bertani (LB) medium (10 g l^-1^ NaCl, 5 g l^-1^ yeast extract, 10 g l^-1^ tryptophan, and 10 g l^-1^ agar) and incubated at 28°C overnight. To propagate the liquid bacterial culture, a single colony was taken from the bacterial culture using a loop and transferred to 50 ml of liquid LB medium containing 50 mg l^-1^ kanamycin. This liquid bacterial culture was incubated overnight at 28°C in a shaker at 200 rpm. The density of the incubated bacterial solution was measured using a spectrophotometer and adjusted to an optical density at 600 nm (OD_600_) of 0.5. Acetosyringone was added to the bacterial solution in LB medium at a concentration of 200 µM l^-1^, and the culture was incubated at 200 rpm for 3h to 4h. The explants were then transferred to the prepared EHA105 culture for 20 min and drained on sterile filter paper. Afterward, the explants were incubated in a co-culture medium (consisting of MS medium supplemented with 1 g l^-1^ MES, 1.5 mg l^-1^ BA, 1.5 mg l^-1^ ABA, 200 µM acetosyringone, 3% sucrose, and 7-g plant agar) for 3 days at 28°C. At the end of the 3-day co-culture period, the explants were transferred to a selection medium (consisting of MS medium supplemented with 1.5 mg l^-1^ BAP, 1.5 mg l^-1^ ABA, 1 mg l^-1^ phosphonitricine [PPT], 3% sucrose, and 7 g agar) and incubated at 26°C with a 16h photoperiod until shoot formation occurred. Also, mediums were supplemented with 300 mg l^-1^ timentin to remove EHA105 until acclimatization after the co-cultivation. The formed shoots were separated from the explants and placed in a shoot development medium (consisting of MS medium supplemented with 1 mg l^-1^ GA_3_, 100 mg l^-1^ timentin, 0.25 mg l^-1^ PPT, 3% sucrose, and 7 g plant agar) to promote elongation. Plantlets displaying healthy shoot growth were then rooted in a medium consisting of ½ MS medium containing 1 mg l^-1^ IBA, 50 mg l^-1^ timentin, 3% sucrose, and 7 g plant agar. After the acclimatization process, regenerated G27 and G247 were transferred to a greenhouse and their DNAs were extracted using CTAB method.

### Identification of *elF4E* mutations

The positions of gRNA in the G27 and G247 plants were amplified using PCR and primers MC1F/R (521) and MC2F/R (451 bp). The PCR reaction was prepared using Dream Taq 2× Master Mix and carried out with pre-denaturation at 95°C for 2 min, 35 cycles of denaturation at 95°C for 20 s, annealing at 56°C for 30 s, and extension at 72°C for 1 min followed by a final extension at 72°C for 5 min. The Cas9 cleavage sites of the non-edited G27 and G247 *elF4E*’s gRNA1 and gRNA2 positions contain *MvaI* and *PsuI* restriction enzyme sites, respectively. These restriction enzymes were used to cut only the non-edited elF4E fragment and detect allelic mutations in T0, T1, and F1 generations. The restricted fragments were visualized on a 2% agarose gel after electrophoresis. The expected fragment sizes for non-edited plants were 293 and 228 bp for the elF4E-gRNA1 fragment and 295 and 156 bp for the elF4E-gRNA2 fragment, while the non-restricted fragments (521 bp for elF4E-gRNA1 and 451 bp for elF4E-gRNA2) were considered to putative mutants due to loss of the restriction site as a result of NHEJ after Cas9 cleavage. The gRNA targets of the mutant G27 and G247 plants were amplified using PCR and sequenced using the Sanger method. The resulting data were analyzed using Geneious Prime to identify mutations in the *elF4E* gene. The presence or absence of introns and potential changes in amino acid sequence were also analyzed. Homozygous mutants were selected from the T0 generation and self-pollinated to produce seeds. The presence of T-DNA was confirmed using PCR in the T1 generation, and transgene-free mutants were selected. Mutation analysis was also conducted in the F1 generation after crossing T2 G27 and G247 mutants.

### Crossing plots

T2 seeds were obtained from homozygous mutant parental lines in the T1 generation that were free of transgenes. These seeds were germinated, and silver nitrate was applied to the apical meristems of both mutant G247 plants and non-edited G247 plants to induce male flower formation. Male flowers were collected, and their stamens were used to pollinate the pistils of female flowers from the mutant G27 plant. Both mutant and non-edited G247 lines were crossed with the mutant G27 line, with the different combinations indicated in **Table 1**. These crossing combinations were designed to produce F1 plants that were heterozygous or homozygous for the mutation, as well as F1 plants that were not edited. Seeds were harvested from these crosses and germinated for further analysis, including virus inoculation, mutation testing, and morphological characterization.

### Virus inoculation

A phosphate buffer (pH 7.5) was prepared by mixing 2% potassium phosphate (K_2_HPO_4_), 0.1% sodium sulfite (Na_2_SO_3_), and 0.01% β-mercaptoethanol. This buffer was used to create an inoculum for ZYMV, WMV, and PRSV for F1 plants. The viruses were collected from infected cucumber plants in a greenhouse, ground in a 0.02 M phosphate buffer, and used to mechanically inoculate 20 plants, including five wild-type control F1 plants. The inoculation was carried out using a sponge pad soaked in the buffer solution containing the virus, and the inoculated plants were observed over time. Disease symptoms were evaluated at 7–10 days post-inoculation (dpi) for WMV and ZYMV and at 3 weeks post-inoculation for PRSV. Inoculated plants were scored using different scales: 0 to 4 (0 = no symptoms and 4 = severe mosaic on many leaves) for PRSV (Wai and Grumet, 1995), 1 to 9 (1 = no symptoms and 9 = plant dead) for ZYMV (Guner et al., 2018), and 0 to 5 (0 = no symptoms and 5 = severe mosaic and leaf distortion) for WMV (Palomares-Rius et al., 2011). Vector-transmission inoculation was carried out using *Aphis gossypii*, with approximately 8–10 aphids per plant (Gal-On et al., 1992).

### Detection of inoculated viruses and viral loads

RT-PCR and qRT-PCR were performed to detect and quantify the accumulation of ZYMV, WMV, and PRSV in non-edited and mutant F1 plants. Total RNA was extracted from the leaf discs of the plants using a Total RNA Isolation Kit (Thermo Fisher Scientific, Karlsruhe, Germany) according to the manufacturer’s protocol. The purified RNA was diluted in nuclease-free water, and 1 ng of RNA was used in the RT-PCR reaction. The reaction mix was prepared with 1 µl of Verso Enzyme Mix, 25 µl of 2× 1-Step PCR ReddyMix, 2.5 µl of RT Enhancer, and 7 µl of nuclease-free water. ZYMV-CP-285F_LK/ZYMV-CP-782R_LK (498 bp), PRSV-F/R (~950 bp), and WMV-F/R (535 bp) were used to detect ZYMV, PRSV, and WMV, respectively, and their sequences are in **Supplementary Table S7** (Valekunja et al., 2016; Kaldis et al., 2018; de Souza Aguiar et al., 2019). The RT-PCR was performed under the following conditions: cDNA synthesis at 50°C for 15 min, RT inactivation at 95°C for 2 min, 35 cycles (denaturation at 95°C for 20 s, annealing at 50°C [ZYMV], 52°C [WMV], and 58°C [PRSV] for 30 s, extension at 72°C for 1 min), and final extension at 72°C for 5 min. The RT-PCR products were analyzed by electrophoresis on an agarose gel (1.5%) and visualized using a UV transilluminator.

The detection of ZYMV, PRSV, and WMV in plant samples was performed using DAS-ELISA kits from Agdia, which contained IgG and conjugate antibodies, as well as positive and negative controls. A mixture of 100 ml of coating buffer and 1 µl of IgG antibodies was prepared according to the manufacturer’s instructions for a 1/100 dilution and distributed to the wells for a total of 10 samples. The mixture was incubated at 37°C for 4h, washed with washing buffer, and dried. Plant tissue samples for virus inoculation included six mutant plants, one wild-type plant, and positive and negative controls. According to the protocol, 100 mg of leaf tissue was taken from each virus-infected plant sample, crushed with 600 µl of extraction buffer in a mortar and pestle, and mixed with positive and negative controls from Agdia (Elkhart, Indiana, USA). One hundred of microliter of the crushed mixture was added to the wells and incubated in the refrigerator at 4°C overnight. The next day, after washing and drying with washing buffer, a mixture of conjugate buffer and antibody was prepared in the same proportions as the IgG antibodies and added to the wells. After incubating for 2h at 37°C, a mixture of PNP and substrate buffer was prepared, and 100 µl was added to the wells. The results were observed using an ELISA reader and samples, with an absorbance value at 405 nm greater than two times the negative control was considered positive and the results were recorded for statistical analysis. This process was repeated three times separately for each virus.

Isolated RNAs were used in qRT-PCR to determine the viral load for each genotype. The iTaq Universal SYBR Green One-Step Kit (Bio-Rad, California, USA) was used to prepare the reaction mixture according to the manufacturer’s protocol, and qRT-PCR primers were given in **Supplementary Table S7**. qRT-PCR was performed under the following conditions: cDNA synthesis at 50°C for 10 min, RT inactivation at 95°C for 1 min, 40 cycles of 95°C for 10 s, and 30°C for 60 s. All reactions were done with three replicates for each sample in CFX96 Touch (Bio-Rad). Relative viral accumulation was determined for each genotype using the 2^−ΔΔCT^ method.

### Statistical analyses and data visualization

The length of internodes, leaves, and fruits were measured in mature plants, and the number of fruits and fruit weight was also determined as yield criteria in mutant F1s (M36 × M4464) and non-edited F1 plants. Quantitative data were collected from 10 edited and non-edited F1 plants for each criterion (**Supplementary Table S5**). R version 4.2.2 was used for Welch Two Sample *t*-test and constitution of boxplots graphs with “ggplot2” package. “tidyverse”, “dplyr”, and “multcompView” packages were used for the quantification of viral accumulation determined by qRT-PCR and ELISA and data visualization. Evaluation of inoculated ZYMV, WMV, and PRSV symptoms data were performed in Microsoft Excel.

## Data availability statement

The original contributions presented in the study are included in the article/**Supplementary Material**. Further inquiries can be directed to the corresponding author.

## Author contributions

Conceptualization: HF and OC. Data curation: EA, HF, and OC. Formal analysis: PS, AI, YO, and GF. Funding acquisition: HF and AA. Methodology: HF, OC, EA, GF, and AI. Statistical analysis: MT. Project administration: HF. Resources: HF and AA. Supervision: HF, OC, and EA. Writing – original draft: MT. Writing – review and editing: OC. All authors contributed to the article and approved the submitted version.
