# Supplementary material for: Knockout of elF4E using CRISPR/Cas9 for large-scale production of resistant cucumber cultivar against WMV, ZYMV, and PRSV
Source: Front Plant Sci. 2023 Mar 17;14:1143813. doi: 10.3389/fpls.2023.1143813 (PMC10064079; doi:10.3389/fpls.2023.1143813)
Supplement: Supplementary file 1 [file DataSheet_1.docx]

Supplementary Material

Using CRISPR/Cas9 to knockout elF4E for large-scale production of resistant cucumber cultivars against WMV, ZYMV, and PRSV

Hakan Fidan^1^, Ozer Calis^1*^ Esin Ari^2^, Aydin Atasayar^3^, Pelin Sarikaya^1,3^, Mumin Ibrahim Tek^1^, Ahmet Izmirli^2^, Yasemin Oz^2^ and Gulsah Firat^3^

^1^ Plant Protection Department Faculty of Agriculture Akdeniz University, Antalya, Türkiye

^2^ Agricultural Biotechnology Department, Faculty of Agriculture, Akdeniz University, Antalya, Türkiye

^3^ AD ROSSEN Seeds, Antalya, Türkiye

* Correspondence: Ozer Calis: [ozercalis@akdeniz.edu.tr](mailto:ozercalis@akdeniz.edu.tr)


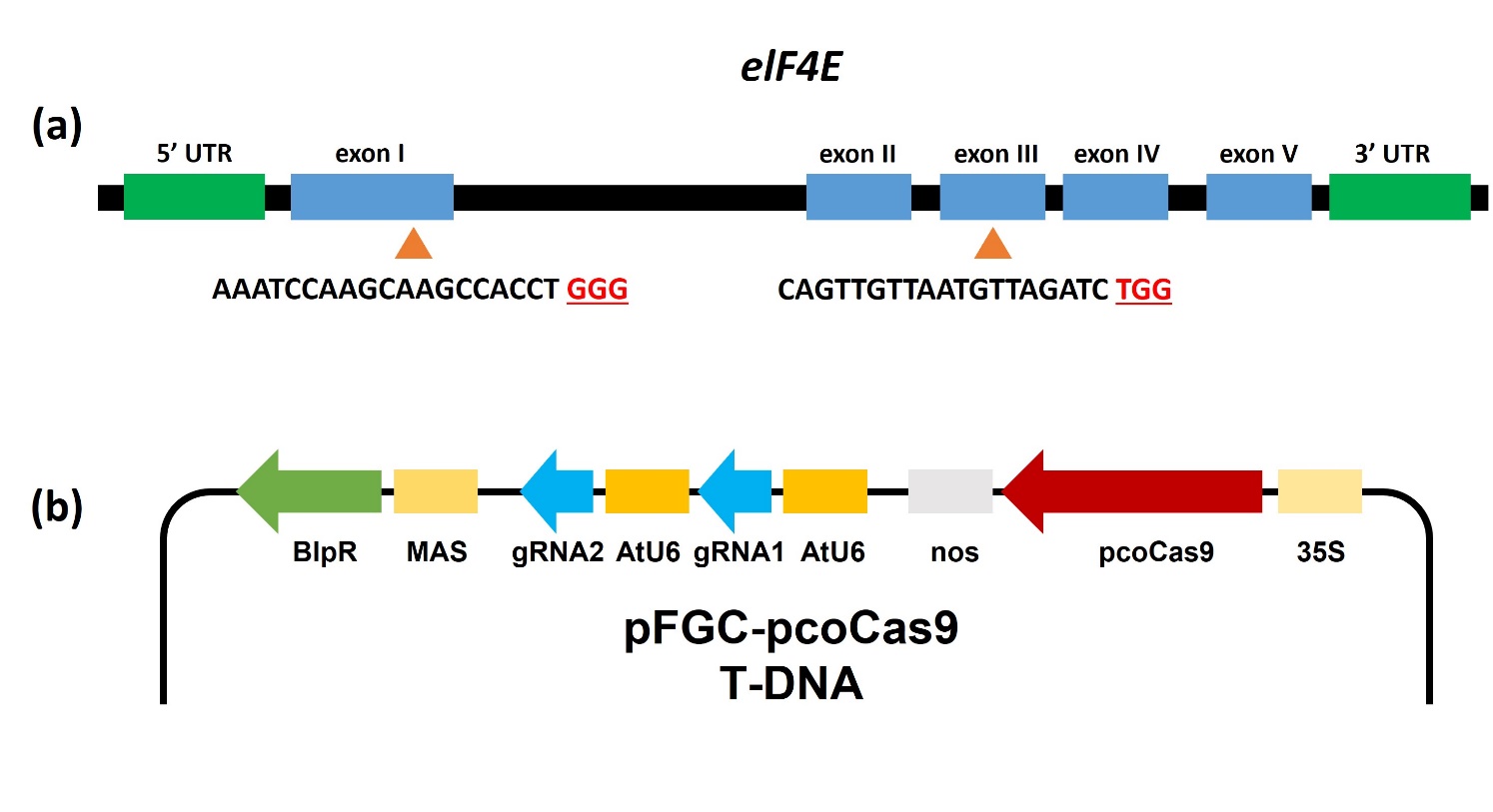


**Supplementary Figure 1.** Schematic representation of gene editing of eukaryotic translation initiation factor (eIF4E) gene in transgenic cucumber plants using the CRISPR/Cas9. (a) Cas9/sgRNA constructs targeted the eIF4E gene. The figure includes a cucumber eIF4E genomic map with sgRNA1 and sgRNA2 target sites located in exon 1 and exon III, respectively. The target sequences are shown with the protospacer adjacent motif (PAM) marked in red and underlined. (b) The pFGC-pcoCas9 plasmid contains; BlpR (phosphinothricin acetyltransferase, confers resistance to bialaphos or phosphinothricin), MAS (mannopine synthase promoter), gRNA (guide RNA), AtU6 (Arabidopsis U6 promoter), nos (nopaline synthase terminator and poly(A) signal), pcoCas9 (plant-codon optimized Cas9), and 35S promoter


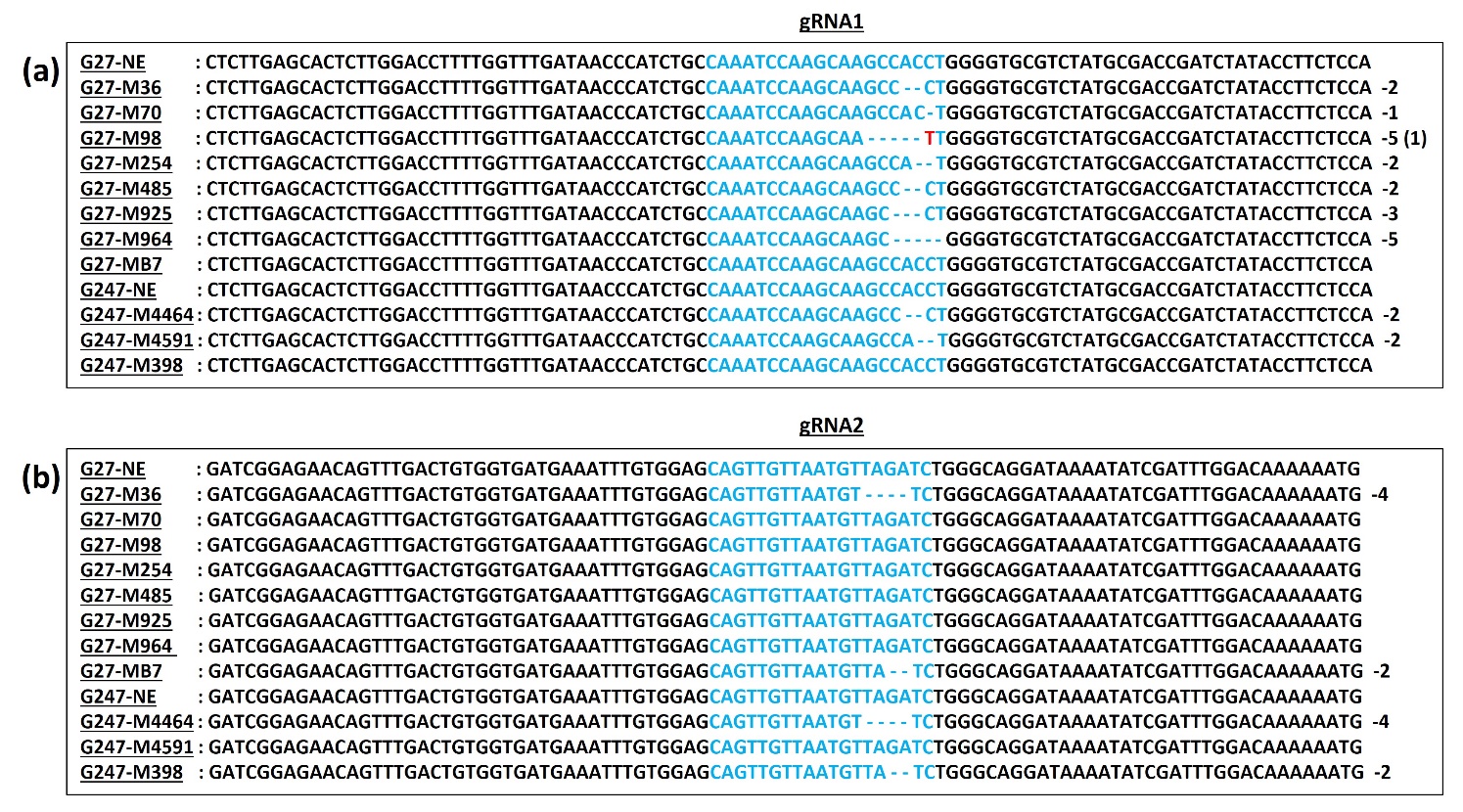


**Supplementary Figure 2.** Alignment of *elF4E* target sequences of eight T2 G27 and three T2 G247 regenerated cucumber plants with the G27-NE and G247-NE for eIF4E. deletions in gRNA1 (a) and gRNA2 (b) target are indicated by blue dashes, and the sizes of the deletions in nucleotides are marked on the right side of the sequence.

**Supplementary Table 1.** Optimization of transformation protocol for *Agrobacterium tumefaciens* strain, pre-culture, and seedling-age and regenerated shoots of G27 and G247

| **Trial** | **Sedds** | | **Seedling**  **age (day)** | **Number of explants** | | | | **pre-culture** | **strain** | **Number of regenerated shoots** | | | | **regenerated shoots %** | | | | |
| --- | --- | --- | --- | --- | --- | --- | --- | --- | --- | --- | --- | --- | --- | --- | --- | --- | --- | --- |
|  |  |  |  | **G27** | | **G247** | |  |  | **G27** | | **G247** | | **G27** | | **G247** | |  |
|  | **G27** | **G247** |  | **C** | **H** | **C** | **H** |  |  | **C** | **H** | **C** | **H** | **C** | **H** | **C** | **H** |  |
| T1 | 40 | 40 | 5 | 120 | 40 | 140 | 40 | + | LBA4404 | 0 | 1 | 0 | 0 | 0.00 | 2.50 | 0.00 | 0.00 |  |
| T2 | 27 | 29 | 5 | 81 | 27 | 87 | 29 | + | LBA4404 | 0 | 0 | 0 | 0 | 0.00 | 0.00 | 0.00 | 0.00 |  |
| T3 | 29 | 29 | 5 | 85 | 29 | 86 | 29 | + | LBA4404 | 0 | 0 | 0 | 0 | 0.00 | 0.00 | 0.00 | 0.00 |  |
| T4 | 30 | 30 | 5 | 90 | 30 | 95 | 30 | + | LBA4404 | 0 | 0 | 0 | 0 | 0.00 | 0.00 | 0.00 | 0.00 |  |
| T5 | 39 | 40 | 5 | 117 | 39 | 120 | 40 | + | LBA4404 | 5 | 0 | 2 | 0 | 4.27 | 0.00 | 1.67 | 0.00 |  |
| T6 | 39 | 38 | 5 | 115 | 39 | 120 | 38 | + | EHA105 | 0 | 0 | 0 | 0 | 0.00 | 0.00 | 0.00 | 0.00 |  |
| T7 | 50 | 50 | 5 | 150 | 50 | 160 | 50 | + | EHA105 | 0 | 0 | 4 | 1 | 0.00 | 0.00 | 2.50 | 2.00 |  |
| T8 | 50 | 50 | 5 | 140 | 50 | 155 | 50 | + | EHA105 | 0 | 0 | 3 | 0 | 0.00 | 0.00 | 1.94 | 0.00 |  |
| T9 | 70 | 70 | 5 | 200 | 70 | 210 | 70 | + | EHA105 | 0 | 1 | 0 | 0 | 0.00 | 1.43 | 0.00 | 0.00 |  |
| T10 | 75 | 75 | 5 | 220 | 75 | 225 | 75 | + | EHA105 | 0 | 0 | 0 | 0 | 0.00 | 0.00 | 0.00 | 0.00 |  |
| T11 | 70 | 70 | 5 | 200 | 70 | 220 | 70 | + | EHA105 | 0 | 0 | 0 | 0 | 0.00 | 0.00 | 0.00 | 0.00 |  |
| T12 | 75 | 75 | 5 | 322 | 75 | 276 | 75 | - | EHA105 | 2 | 2 | 1 | 7 | 0.62 | 2.67 | 0.36 | 9.33 |  |
| T13 | 70 | 70 | 5 | 180 | 70 | 154 | 70 | - | EHA105 | 0 | 0 | 1 | 5 | 0.00 | 0.00 | 0.65 | 7.14 |  |
| T14 | 68 | 70 | 5 | 191 | 68 | 160 | 70 | - | EHA105 | 0 | 0 | 0 | 0 | 0.00 | 0.00 | 0.00 | 0.00 |  |
| T15 | 70 | 69 | 5 | 169 | 70 | 178 | 69 | - | EHA105 | 0 | 0 | 0 | 0 | 0.00 | 0.00 | 0.00 | 0.00 |  |
| T16 | 70 | 60 | 5 | 211 | 70 | 220 | 60 | - | EHA105 | 0 | 0 | 0 | 0 | 0.00 | 0.00 | 0.00 | 0.00 |  |
| T17 | 58 | 59 | 5 | 179 | 58 | 191 | 59 | - | EHA105 | 0 | 0 | 0 | 0 | 0.00 | 0.00 | 0.00 | 0.00 |  |
| T18 | 55 | 60 | 5 | 169 | 55 | 219 | 60 | - | EHA105 | 0 | 0 | 0 | 0 | 0.00 | 0.00 | 0.00 | 0.00 |  |
| T19 | 60 | 60 | 5 | 103 | 60 | 167 | 60 | - | EHA105 | 0 | 0 | 0 | 0 | 0.00 | 0.00 | 0.00 | 0.00 |  |
| T20 | 72 | 72 | 5 | 258 | - | 230 | - | - | EHA105 | 8 | - | 19 | - | 3.10 | - | 8.26 | - |  |
| T21 | 80 | 80 | 5 | 193 | - | 291 | - | - | EHA105 | 0 | - | 23 | - | 0.00 | - | 7.90 | - |  |
| T22 | 50 | 55 | 5 | 174 | - | 162 | - | - | EHA105 | 0 | - | 20 | - | 0.00 | - | 12.35 | - |  |
| T23 | 50 | 50 | 1 | 102 | - | 107 | - | - | EHA105 | 6 | - | 21 | - | 5.88 | - | 19.63 | - |  |
| T24 | 50 | 50 | 1 | 92 | - | 130 | - | - | EHA105 | 20 | - | 14 | - | 21.74 | - | 10.77 | - |  |
| T25 | 50 | 50 | 1 | 83 | - | 97 | - | - | EHA105 | 5 | - | 16 | - | 6.02 | - | 16.39 | - |  |
| T26 | 75 | 75 | 1 | 134 | - | 141 | - | - | EHA105 | 10 | - | 16 | - | 7.46 | - | 11.35 | - |  |
| T27 | 54 | 54 | 1 | 105 | - | 148 | - | - | EHA105 | 5 | - | 22 | - | 4.76 | - | 14.86 | - |  |
| T28 | 100 | 100 | 1 | 178 | - | 196 | - | - | EHA105 | 8 | - | 43 | - | 4.49 | - | 21.94 | - |  |
| T29 | 50 | 50 | 1 | 103 | - | 95 | - | - | EHA105 | 34 | - | 32 | - | 33.01 | - | 33.68 | - |  |
| T30 | 50 | 50 | 1 | 267 | - | 97 | - | - | EHA105 | 68 | - | 15 | - | 25.47 | - | 15.46 | - |  |
| **Total** | **1.832** | **1730** | NA | **4.731** | **1.045** | 4.877 | 1.044 | NA | NA | 171 | 4 | 252 | 13 | NA | NA | NA | NA |  |
| **Average** | **61.1** | **57.7** | NA | **157.7** | **55.0** | 162.6 | 54.9 | NA | NA | 5.7 | 0.2 | 8.4 | 0.7 | 3.9 | 0.3 | 6.0 | 1.0 |  |

C: cotyledon, H: hypocotyl, NA: not applicable

**Supplementary Table 2.** Number of regenerated shoots with optimized protocol

| **Transformation no** | **Seeds** | | **Explant** | | **Number of Regenerated Shoots** | | **Regenerated Shoots (%)** | |
| --- | --- | --- | --- | --- | --- | --- | --- | --- |
|  | **G27** | **G247** | **G27** | **G247** | **G27** | **G247** | **G27** | **G247** |
| **T23** | 50 | 50 | 102 | 107 | 6 | 21 | 5.88 | 19.63 |
| **T24** | 50 | 50 | 92 | 130 | 20 | 14 | 21.74 | 10.77 |
| **T25** | 50 | 50 | 83 | 97 | 5 | 16 | 6.02 | 16.49 |
| **T26** | 75 | 75 | 134 | 141 | 10 | 16 | 7.46 | 11.35 |
| **T27** | 60 | 54 | 105 | 148 | 5 | 22 | 4.76 | 14.86 |
| **T28** | 100 | 100 | 178 | 196 | 8 | 43 | 4.49 | 21.94 |
| **T29** | 50 | 50 | 103 | 95 | 34 | 32 | 33.01 | 33.68 |
| **T30** | 150 | 50 | 267 | 97 | 68 | 15 | 25.47 | 15.46 |
| **Average** | **73.1** | **59.9** | **133.0** | **126.4** | **19.5** | **22.4** | **13.6** | **18.0** |
| **Total** | **585** | **479** | **1064** | **1011** | **156** | **179** | **-** | **-** |

S**upplementary Table 3.** Number of regenerated plats and segregation of transgenic and transgene-free plants in T1 generation

|  |  | T0 | | | | |  | | T1 | | | | | |  |
| --- | --- | --- | --- | --- | --- | --- | --- | --- | --- | --- | --- | --- | --- | --- | --- |
|  |  | G27 |  | G247 |  | Total | |  | | G27 |  | G247 |  | Total | |
| No. of regenerated |  | 38 |  | 24 |  | 62 | |  | | NA |  | NA |  | NA | |
| No. Transgenic |  | 34 |  | 22 |  | 56 | |  | | 2315 |  | 1639 |  | 3954 | |
| No. Expected Transgenic |  | NA |  | NA |  | NA | |  | | 2299.50 |  | 1581.75 |  | 3881.25 | |
| No. Transgene-free |  | NA |  | NA |  | NA | |  | | 751 |  | 470 |  | 1221 | |
| No. Expected Transgene-free |  | NA |  | NA |  | NA | |  | | 766.50 |  | 527.25 |  | 1293.75 | |

(p<0.05; ꭕ2=9.49)

**Supplementary Table 4.** Number of selected transgene-free mutant plants and their mutation types in T1 generation

|  |  | T1 | | | | |  | | Mutation Types | | | | | |  |
| --- | --- | --- | --- | --- | --- | --- | --- | --- | --- | --- | --- | --- | --- | --- | --- |
|  |  | G27 |  | G247 |  | Total | |  | | deletion |  | insertion |  | substitution | |
| **No. of examined plants with PCR** |  | 251 |  | 106 |  | 357 | |  | | NA |  | NA |  | NA | |
| **Hom. Mutant for gRNA1** |  | 42 |  | 16 |  | 58 | |  | | NA |  | NA |  | NA | |
| **Het. Mutant for gRNA1** |  | 68 |  | 24 |  | 92 | |  | | NA |  | NA |  | NA | |
| **Hom. Mutant for gRNA2** |  | 29 |  | 12 |  | 41 | |  | | NA |  | NA |  | NA | |
| **Het. Mutant for gRNA2** |  | 39 |  | 16 |  | 55 | |  | | NA |  | NA |  | NA | |
| **Hom. Mutant for gRNA1/gRNA2** |  | 6 |  | 4 |  | 10 | |  | | NA |  | NA |  | NA | |
| **Het. Mutant for gRNA1/ gRNA2** |  | 8 |  | 7 |  | 15 | |  | | NA |  | NA |  | NA | |
| **Non-edited** |  | 59 |  | 27 |  | 86 | |  | | NA |  | NA |  | NA | |
| **No. of sequenced plants** |  | 20 |  | 20 |  | 40 | |  | | 1 bp, 2 bp, 3 bp, 4 bp, 5 bp |  |  |  | C→T | |
| **Mutations at only gRNA1** |  | 9 |  | 5 |  | 14 | |  | | 1 bp, 2 bp, 3 bp, 5 bp |  | - |  | C→T | |
| **Mutations at only gRNA2** |  | 6 |  | 3 |  | 9 | |  | | 2 bp, 4 bp |  | - |  | - | |
| **Mutations at gRNA1 and gRNA2** |  | 3 |  | 2 |  | 5 | |  | | 2 bp, 4 bp |  | - |  | - | |

S**upplementary Table 5.** Comparing yield and qualitative criteria in edited (M36 x M4464) and non-edited (G27 x G247) F1 plants

|  | **Internode Length (cm)** | | **Plant Lenght (cm)** | | **Leaf Lenght (cm)** | | **Yield per Plant (g)** | | **Single Fruit Weight (g)** | | **Fruit Length (cm)** | |
| --- | --- | --- | --- | --- | --- | --- | --- | --- | --- | --- | --- | --- |
| # | **G27 x G247** | **M36 x M4464** | **G27 x G247** | **M36 x M4464** | **G27 x G247** | **M36 x M4464** | **G27 x G247** | **M36 x M4464** | **G27 x G247** | **M36 x M4464** | **G27 x G247** | **M36 x M4464** |
| #1 | 8.1 | 8.4 | 220 | 218 | 29 | 30 | 4436 | 4430 | 152 | 151 | 17.5 | 18.4 |
| #2 | 9.5 | 8.9 | 214 | 220 | 30.5 | 29.5 | 4427 | 4422 | 151 | 150 | 18.2 | 18.6 |
| #3 | 9.3 | 9.7 | 215 | 217 | 29 | 30 | 4399 | 4419 | 147 | 149 | 18 | 17.9 |
| #4 | 9.7 | 9.3 | 218 | 219 | 28.5 | 30 | 4436 | 4456 | 149 | 151 | 18.6 | 18.2 |
| #5 | 8.5 | 9.8 | 215 | 214 | 30 | 30 | 4436 | 4452 | 150 | 152 | 18.9 | 18.0 |
| #6 | 8.6 | 8.6 | 218 | 221 | 32 | 31 | 4418 | 4409 | 148 | 146 | 17.8 | 18.6 |
| #7 | 8.4 | 8.4 | 214 | 215 | 29.5 | 29.5 | 4421 | 4440 | 148 | 150 | 19 | 19.1 |
| #8 | 9.5 | 9.2 | 221 | 219 | 31 | 29.5 | 4403 | 4439 | 147 | 150 | 18.4 | 18.6 |
| #9 | 9.8 | 9.4 | 214 | 220 | 30 | 31 | 4418 | 4421 | 147 | 147 | 18.9 | 18.3 |
| #10 | 9.2 | 9.6 | 217 | 218 | 30 | 31 | 4409 | 4419 | 147 | 149 | 17.6 | 18.3 |
| Average | 9.06 | 9.13 | 214.6 | 218.1 | 29.95 | 30.15 | 4420.3 | 4430.7 | 148.6 | 149.5 | 18.29 | 18.4 |
| STD | 0.6058969 | 0.5271517 | 5.621388 | 2.233582 | 1.039498 | 0.6258328 | 13.64673 | 15.50663 | 1.837873 | 1.840894 | 0.5566766 | 0.3464102 |

STD: Standard deviation

S**upplementary Table 6.** Welch Two Sample t-test for comparison of morphological and yield data of edited (M36 x M4464) and non-edited (G27 x G247) F1 plants

| **Variable** |  | ***t*^a^** |  | ***df^b^*** |  | ***p-value*^c^** |  | **95% confidence interval** |
| --- | --- | --- | --- | --- | --- | --- | --- | --- |
| plant length |  | -1.8298 |  | 11.773 |  | 0.09271 |  | -7.6766251  0.6766251 |
| leaf length |  | -0.52125 |  | 14.767 |  | 0.60990 |  | -1.018954  0.618954 |
| internode length |  | -0.27562 |  | 17.662 |  | 0.78600 |  | -0.6043008  0.4643008 |
| yield per plant |  | -1.5921 |  | 17.714 |  | 0.12900 |  | -24.139425  3.339425 |
| single fruit weight |  | -1.0941 |  | 18 |  | 0.28830 |  | -2.6282136  0.8282136 |
| fruit length |  | -0.53054 |  | 15.061 |  | 0.60350 |  | -0.551773  0.331773 |

^a^: t value/score, ^b^ : degrees of freedom; ^c^ : 0.05

**Supplementary Table 7.** Oligonucleotides used in present study.

| Primer | Sequence (5’-3’) |
| --- | --- |
| WMV-F | TTRTTGTTGAATGCTGTCCT |
| WMV-R | GCTGCACAAATTGCCTCAG |
| PRSV-F | GCGGATCCATGTCCAAAAATGAAGCTGTGGAT |
| PRSV-R | GCAAGCTTGTTGCGCATACCCAGGAGAGAGTG |
| ZYMV-CP-285F_LK | GGGGATCCAGCGTCTCATCAGCAATTCG |
| ZYMV-CP-782R_LK | GGGGATCCGTGTGCCGTTCAGTGTCTTC |
| WMV-HP-RVL-F | GCGTGCTTTATGAATGCTCA |
| WMV-HP-RVL-R | CTGACAAAACCTCGCACTGA |
| PRSV -CP-QR-F | CCCAGAATTAAGGGGAAAGC |
| PRSV -CP-QR-R | CTGGGGATGTACCATTTTCG |
| ZYMV-RQVL-CP-F | AGGAGTTTGGCACGATATGC |
| ZYMV-RQVL-CP-R | TGGCAACATTTCCATCAAGA |
| MC1F | ACGATCAAAGCTACATCGGC |
| MC1R | TCTCCAGCCCTCACATTCAT |
| MC2F | ATCCTGTTTGTGCGAGTGGA |
| MC2R | GCCACTGCCAGAATGAAGTA |
| Ag-CT0-F | AAATCCAAGCAAGCCAC |
| Ag-CT0-R | GATCTAACATTAACAACTGAATC |
